# Has *Chiranjeevi Yojana* changed the geographic availability of free comprehensive emergency obstetric care services in Gujarat, India?

**DOI:** 10.3402/gha.v8.28977

**Published:** 2015-10-06

**Authors:** Kranti Suresh Vora, Sandul Yasobant, Amit Patel, Ashish Upadhyay, Dileep V. Mavalankar

**Affiliations:** 1 Indian Institute of Public Health – Gandhinagar, Ahmedabad, India; 2 School of Policy, Government, and International Affairs, George Mason University, Fairfax, VA, USA

**Keywords:** maternal mortality, India, Gujarat, CEmOC, 2FCA, public private partnership, *Chiranjeevi Yojana*, GIS

## Abstract

**Background:**

The high rate of maternal mortality in India is of grave concern. Poor rural Indian women are most vulnerable to preventable maternal deaths primarily because they have limited availability of affordable emergency obstetric care (EmOC) within reasonable geographic proximity. Scarcity of obstetricians in the public sector combined with financial barriers to accessing private sector obstetrician services preclude this underserved population from availing lifesaving functions of comprehensive EmOC such as C-section. In order to overcome this limitation, Government of Gujarat initiated a unique public–private partnership program called *Chiranjeevi Yojana* (CY) in 2005. The program envisaged leveraging private sector providers to increase availability and thereby accessibility of EmOC care for vulnerable sections of society. Under CY, private sector providers render obstetric care services to poor women at no cost to patients. This paper examines the CY's effectiveness in improving availability of CEmOC services between 2006 and 2012 in three districts of Gujarat, India.

**Methods:**

Primary data on facility locations, EmOC functionality, and obstetric bed availability were collected in the years 2012 and 2013 in three study districts. Secondary data from Census 2001 and 2011 were used along with required geographic information from Topo sheets and Google Earth maps. ArcGIS version 10 was used to analyze the availability of services using two-step floating catchment area (2SFCA) method.

**Results:**

Our analysis suggests that the availability of CEmOC services within reasonable travel distance has greatly improved in all three study districts as a result of CY. We also show that the declining participation of the private sector did not result in an increase in distance to the nearest facility, but the extent of availability of providers for several villages was reduced. Spatial and temporal analyses in this paper provide a comprehensive understanding of trends in the availability of EmOC services within reasonable travel distance.

**Conclusions:**

This paper demonstrates how GIS could be useful for evaluating programs especially those focusing on improving availability and geographic accessibility. The study also shows usefulness of GIS for programmatic planning, particularly for optimizing resource allocation.

Reducing maternal mortality ratio (MMR) is important for India, where a high rate of maternal mortality is a major public health issue (1, 2). The majority of maternal deaths in India can be averted by interventions that address the issue of a limited availability of skilled birth attendance and emergency obstetric care (EmOC) (3–5). There are multiple determinants of availability of EmOC in developing countries such as socioeconomic profile of the users, healthcare facilities within reasonable travel distance, and number/capacity of healthcare facilities to provide timely and appropriate services to all (6). Poor rural mothers have limited access to EmOC because of financial and geographic barriers leading to higher maternal mortality among this underserved section of society (4, 7–10). Provision of comprehensive emergency obstetric care (CEmOC) services, which includes blood transfusion and C-section along with the management of common obstetric complications, requires availability of specialists (obstetricians, anesthetists, and pathologists) and infrastructure for blood transfusion services and C-section. Public sector facilities in India have a significant shortfall (67%) of obstetricians in rural areas, resulting in restricted availability of lifesaving obstetric services. There are about 30,000 obstetricians in India, out of which only about 1,600 are in the public sector leading to disparities in both availability and access to CEmOC in public sector (11, 12).

To overcome limited obstetric care availability in the public sector, the Government of India and several state governments have devised policies to make maternal healthcare affordable, available, and accessible to all by collaborating with the private sector. *Chiranjeevi Yojana* (CY) is one such innovative public–private partnership program, implemented in 2006 in the state of Gujarat aimed to improve availability, accessibility, and affordability of CEmOC services for poor women (13). Gujarat, an affluent state in the western part of India, has a severe shortage of obstetricians in the public sector (98% shortfall), leading to limited access to affordable CEmOC, particularly for poor rural women (13–16). On the contrary, there is a strong presence of private obstetricians at the subdistrict levels (17). The program aimed to leverage widely available private obstetricians to improve financial and geographic availability of skilled birth attendance and CEmOC to poor women of Gujarat (18, 19).

Under CY, an accredited private provider offers free delivery care, including CEmOC, to eligible women and receives payment directly from the government for the services rendered. Eligibility criteria for CY benefits include both financial and social parameters: women under the poverty line or women belonging to a socially backward class. The government pays INR 4,000 per delivery (approximately USD 67) for 100 deliveries as a package irrespective of type of delivery services performed, that is, normal delivery or C-section. This block payment mechanism helps to deter private providers from performing more C-sections and claim higher payment from the government. About 1 million births have been covered under CY so far (18). Although the program has been evaluated in the past, to the best of our knowledge, the program's effectiveness in increasing geographic availability of free CEmOC services has not been studied so far (20–23). This paper aims to fill this gap in literature and examine if the program improved the availability of free CEmOC between 2006 and 2012 in the study districts.

Geographic availability of services is measured by studying the location of facilities (supply) with respect to distribution of population (demand). A systematic study of spatial organization of supply and demand could reveal areas that are deprived of services. The geographic disparities in access to services have received considerable attention from planners and researchers as identifying areas with limited geographic availability of healthcare services could allow evidence-based location decisions for new healthcare facilities (24, 25). Thus, an accurate and detailed analysis of geographic availability is an important tool for planning and policy-making, especially in terms of choosing locations for additional healthcare facilities. The main purpose of this paper is to study the spatiotemporal changes in availability of free CEmOC services for the poor/tribal women as a result of this program. The study was done in three primarily rural districts of Gujarat, namely Sabarkantha, Surendranagar, and Dahod for the years 2006–2013.

## Methodology

### Data

Three types of data were collected from a variety of sources for location-specific data on both ‘supply side’ (i.e. providers) and ‘demand side’ in all study districts (i.e. target populations). The data sources used for the analysis were:

1. Village-level population data: the village-level population data were obtained for 2001 and 2011 from Census of India.
2. Geographic data for base map: the Administrative Atlas of the Census of India (2011) provided administrative boundaries for the base map. Information from Topo sheets (1975) and open series maps from Survey of India (2011) were combined to triangulate the road network information. Google Earth imageries (2014) were used to augment relatively old Topo sheets information.
3. Location and capacity of facilities: a facility-based survey was conducted during the year 2012–2013 in Sabarkantha, Surendranagar, and Dahod. There were 99 functional CEmOC facilities surveyed (survey included facilities that had conducted more than 30 deliveries in the past 3 months of the survey date); 93 of them were private sector facilities, whereas the remaining six were public sector facilities. Location and capacity/availability of free CEmOC services were obtained from this survey.

ESRI's ArcGIS^®^ software version 10 was used to prepare the base map and carry out network analysis to obtain travel distances. To this end, the datasets were used to: 1) estimate the population at village level for each year of the study period as a proxy for demand for CEmOC services; 2) calculate the network distance between villages and healthcare facilities using the road network; 3) calculate the summary statistics of average distances travelled; and 4) measure geographic availability of CEmOC from each village using the two-step floating catchment area (2SFCA) method.

### Calculations

#### Population estimates using geometric growth method

For each village, population was obtained for years 2001 and 2011 from Census of India. Dahod had the highest decadal growth (27%) followed by Surendranagar (15%); Sabarkantha only grew by 10% between 2001 and 2011. Understandably, individual village populations grew at different rates as well; some experienced an increase, whereas others experienced a decline. To accurately estimate village-level population for the entire study period, annual growth rates were calculated for each village using village-level decennial population data. Based on the growth rates, population for intermediate years (i.e. from 2002 to 2010) was interpolated and projected for the years 2012 and 2013 using geometric growth method of estimation, which is a widely accepted population estimation method in demography and planning for healthcare resources (26).

#### Base map preparation

ArcGIS was used to first digitize Topo sheets and open series maps into ArcGIS's shape file format from the paper-based hard copy format. Digitized information included village administrative boundaries and road networks required to operationalize 2SFCA method. However, both these sources had some road information missing since they were relatively old maps and did not include information on recently constructed roads and unpaved roads. Using satellite images from the Google Earth (2014), missing information was added to complete the road network in the base map. It was challenging to combine datasets from three different sources especially because both Topo sheets and open source maps are paper maps at a very small scale, whereas Google Earth is a digital map that provides very high-resolution imageries. The topology errors resulting from digitization of paper maps were fixed after visually inspecting and validating the information using the Google Earth maps.

#### Mapping of healthcare facilities

To measure the availability of free CEmOC, facilities that provide free services to all (in case of public sector) or free services to eligible rural poor women (in case of private sector CY) were mapped. All private CEmOC service providers were excluded from the analysis since the purpose was to measure the change in availability that could be attributed to CY. Facilities that were missing the function of assisted vaginal delivery of eight CEmOC functions have been included in the analysis, since a significant proportion of obstetricians in Gujarat do not perform operative vaginal deliveries such as forceps and vacuum. This exception was made because the main objective of the study is to look at the most critical CEmOC services, that is, that of provision of C-section and blood transfusion services. Locations of facilities were collected using hand-held global positioning system (GPS) devices by a team of trained field research assistants. The GPS locations were then geocoded on a base map.

#### Average distance to nearest facility

Once both the demand points, that is, villages and supply locations, of CEmOC service providers were mapped, average travel distance to the nearest CEmOC facility from each village centroid was calculated first. The purpose of this preliminary analysis was to understand the impact of changes in CY participation and its implications on geographic availability of free CEmOC. Using this village-level travel distance to the nearest facility, district-level average travel distance to the nearest facility was obtained for each year. However, the average travel distance to the nearest facility can be misleading as it does not take into account the capacity of service providers and provides comprehensive geographic availability. For example, the nearest facility may be overcrowded because it may be serving a large population with very low capacity, thereby making it less accessible in spite of its geographical proximity. In order to overcome this limitation, the 2SFCA method, which utilizes the capacity of the facility to calculate a single accessibility index (*A*_*i*_) for each village, was used.

#### Two-step floating catchment area method

The 2SFCA has been used to measure spatial accessibility to different health services (27–40). In this paper, the 2SFCA method is used to measure the availability of CEmOC within the specific catchment area for individual villages of three study districts. The analysis is carried out for three different time periods to study temporality. Three different time points are considered for this analysis: 1) year 2006 when CY was first implemented, 2) year 2009 when CY was at peak in terms of participation from private providers, and 3) year 2012 as the end point.

For both steps of the calculation of *A*_*i*_, a reasonable travel distance determines the floating catchment area. The average distance travelled, that is,15 km (as per facility survey) was used as the ‘reasonable’ distance to specify the floating catchment area. To assess the sensitivity of the analysis, a 30 km radius was also tested. For each facility, a floating catchment area was created using 15 km of network distance as a radius first. The population of villages covered in this catchment area was then considered as potential demand points. For simplicity, the population of each village was assumed to be located at the centroid of each village, and the service availability at each facility was calculated in terms of number of beds per 100,000 population within floating catchment area, which is called demand-weighted capacity index of each facility (*C*_*j*_). The capacity index *C*_*j*_ for each facility *j* is calculated by dividing number of beds in facility *j* (*S*_*j*_) with sum of population *P*_*k*_ (in 100,000) for villages *k* that are located within 15 km network distance of the facility *j* as shown in Equation 1.

1 $$C_{j}=S_{j}/\sum P_{k}$$

In the second and final step of the 2SFCA method, *A*_*i*_ was calculated for each village. In this step, the *A*_*i*_ for each village *i* is calculated as a sum of all *C*_*j*_ of the service facilities *j* found within a 15 km radius from the village *i* as shown in Equation 2.

2 $$A_{i}=\sum C_{j}$$

The *A*_*i*_ for village *i* measures the availability of free CEmOC which not only reflects all the facilities available within a threshold travel distance of 15 km but also incorporates the demand-weighted capacity at those facilities. The 2SFCA method provides an accurate measure of availability because it incorporates both the travel distance and demand-weighted capacity of facilities. Finally, using village-level *A*_*i*_, choropleth maps for each of the study districts were prepared for three different time periods to visualize the changes in availability of CEmOC services over time and space.

The ethical permission for conducting facility-level primary survey was obtained from the ethical committee (Ref. 23/2012) of the Indian Institute of Public Health – Gandhinagar (IIPHG). Census data and other geographical information used for the analysis are in the public domain.

## Results

Spatial and temporal analyses include 2,763 villages altogether in three study districts – 1,397 villages of Sabarkantha, 669 villages of Surendranagar, and 697 villages of Dahod. Before the implementation of CY, only public sector facilities provided free services to poor rural women in these areas, which are used as the base case scenario. Only one facility in Surendranagar, two in Dahod, and three in Sabarkantha were providing free CEmOC services in 2006. After implementation of CY in 2006, the number of facilities providing free CEmOC care increased to 12 in Surendranagar, 11 in Dahod, and 32 in Sabarkantha. The number of CY providers varied every year after 2006 in each of these districts, with highest participation in 2009 and lowest in 2012, as shown in Table 1.

**Table 1:** Year-wise availability of free CEmOC facilities for eligible women in three study districts

	Sabarkantha		Dahod		Surendranagar	
			
Year	No. of facilities	No. of obstetric beds	No. of facilities	No. of obstetric beds	No. of facilities	No. of obstetric beds
Pre-CY	3	50	2	30	1	10
2005–2006	31	490	11	148	13	167
2006–2007	32	525	11	148	12	152
2007–2008	38	616	11	148	12	152
2008–2009	43	698	10	118	11	142
2009–2010	44	715	10	118	12	149
2010–2011	47	726	10	118	11	156
2011–2012	49	760	11	129	11	153
2012–2013	21	370	08	77	09	138

Although average availability of obstetric beds increased in all three districts as a result of CY, it was uncertain whether distribution of these services is spread evenly across all areas within these districts. Table 2 shows that the average travel distance to the nearest facility substantially decreased in all three districts after CY was implemented. The average distance to the nearest facility decreased from 38.4 km in 2006 to 15.5 km in 2012 in Sabarkantha, 28.4 km in 2006 to 16.7 km in 2012 in Dahod, and 56.7 km in 2006 to 28.4 km in 2012 in Surendranagar.

**Table 2:** Average distance to the nearest free CEmOC facility (in km)

Year	Sabarkantha	Dahod	Surendranagar
Pre-CY	38.4 (21.5)	28.4 (15.1)	56.7 (23.6)
2005–2006	15.3 (9.1)	17.7 (9.1)	28.3 (17.2)
2006–2007	15.2 (9.2)	17.7 (9.1)	28.5 (17.4)
2007–2008	14.7 (9.2)	17.7 (9.1)	28.4 (17.4)
2008–2009	14.7 (9.2)	17.8 (9.1)	28.4 (17.4)
2009–2010	15.2 (9.2)	17.8 (9.1)	28.3 (17.2)
2010–2011	15.1 (9.2)	17.8 (9.1)	31.1 (17.1)
2011–2012	15.3 (9.2)	15 (8)	28.4 (17.2)
2012–2013	15.5 (9.2)	16.7 (8.5)	28.4 (17.2)

Numbers in parenthesis are standard deviations.

This analysis suggests that the introduction of CY has significantly improved the geographic availability, but changes in the level of CY participation did not change average travel distance over time. One of the reasons could be that additional CY providers participated in urban areas predominantly where other CY providers or a public sector already existed.

Table 3 shows that in Sabarkantha, only 18% of villages had free CEmOC available within a 15 km radius in 2005 which increased to 61% in 2006–2007 after CY implementation. Similarly, in Dahod and Surendranagar, the proportion of villages having a free CEmOC facility within a 15 km radius increased from 20 to 42% and 4 to 22%, respectively. However, it is important to note that more than half of the villages within a 15 km radius in Dahod and Surendranagar still remained without any access to free CEmOC facilities, while villages with facilities within a 15 km radius had improved availability in terms of number of beds. This further indicates that CY providers are geographically concentrated in certain areas. Sensitivity analysis where the distance threshold of catchment area was increased from 15 to 30 km validated findings (Table 4). The areas with no access have remained almost constant throughout this period, suggesting that the change in the number of CY facilities was mainly in the areas where services were already available. These findings are confirmed by the fact that around 2012, despite decline in the numbers of providers the coverage within 15 km did not change significantly (Table 3).

**Table 3:** Percentage of villages with free CEmOC facility within 15 km

Year	Sabarkantha (%)	Dahod (%)	Surendranagar (%)
Pre-CY	18	20.5	3.7
2006–2007	61.1	42.2	22.3
2009–2010	61.3	41.9	22.4
2012–2013	60.3	44.5	22.1

**Fig. 1:**
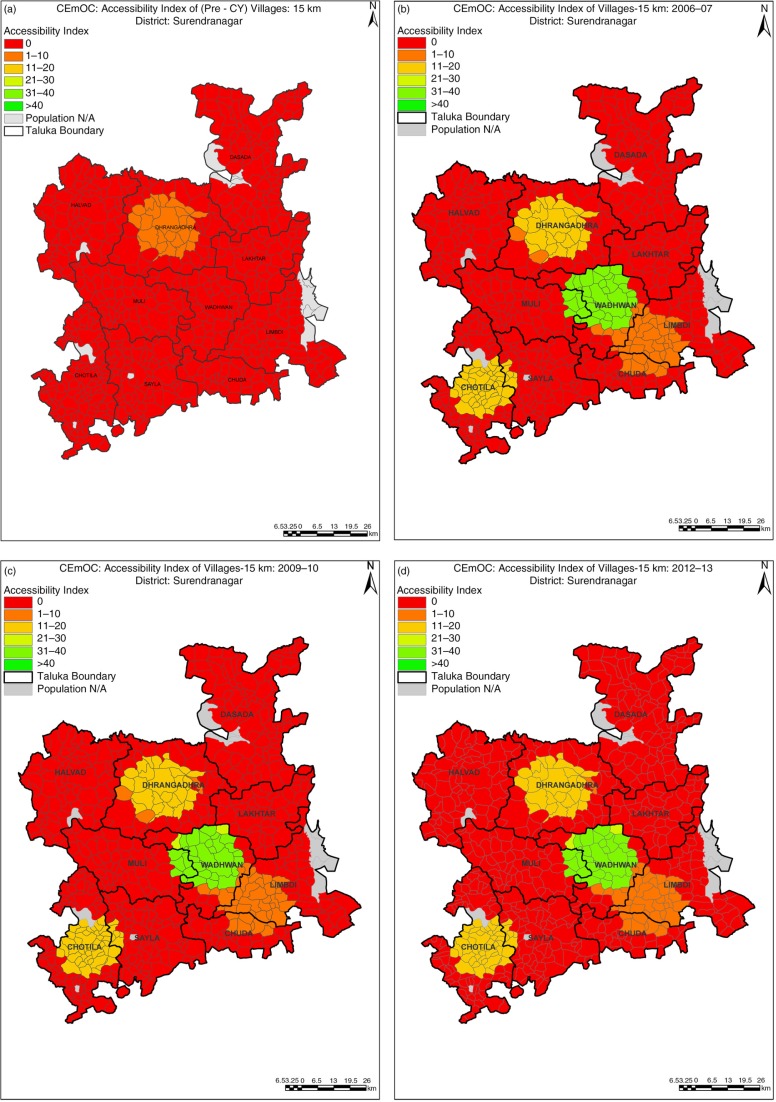
(a–d) Changes in availability in Surendranagar district.

In Surendranagar, *A*_*i*_ increased from less than one bed per 100,000 before implementation of CY to nine beds per 100,000 population in 2006 after CY. It remained the same in 2009 and declined to eight beds per 100,000 population in 2012–2013. The consistently high proportion of villages without any CEmOC facilities within a 15 km radius in Surendranagar (red-colored areas in Fig. 1) suggests that CY did not succeed in improving the availability of CEmOC here. Participation of CY providers did not fluctuate in Surendranagar as much as it did in the other two districts, resulting in an overall lower availability after initial gain compared with other districts.

**Fig. 2:**
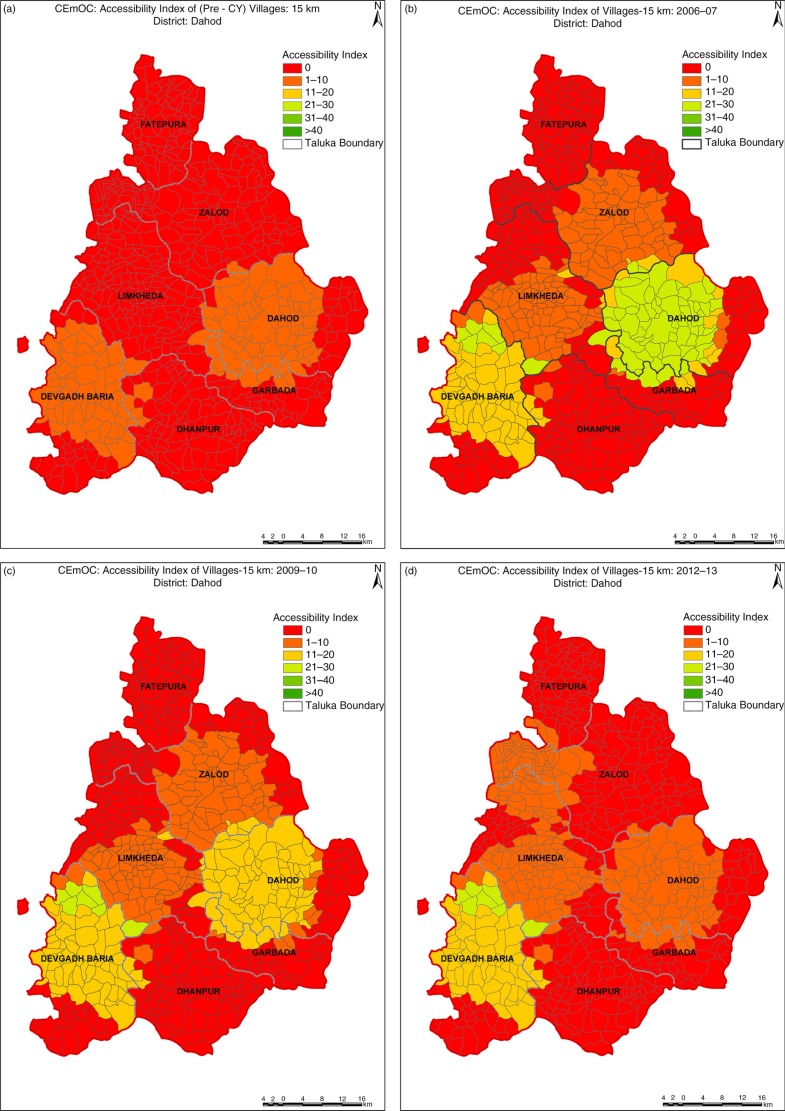
(a–d) Changes in availability in Dahod district.

In Dahod, pre-CY average *A*_*i*_ was two beds per 100,000 population, which increased to eight beds per 100,000 population in 2006. There was a decline from six beds per 100,000 population in 2009–2010 to four beds per 100,000 population in 2012–2013. The upward swing in average *A*_*i*_ initially suggests that the availability and provider choice increased with the higher participation of private providers in CY. The consistently high proportion of villages without any CEmOC within a 15 km radius in Dahod (red-colored areas in Fig. 2) suggests that the number of CY providers gradually declined along with *A*_*i*_, and the proportion of villages with no availability remained relatively constant.

**Table 4:** Percentage of villages with free CEmOC facility within 30 km

Year	Sabarkantha (%)	Dahod (%)	Surendranagar (%)
Pre-CY	59.5	61.8	14.5
2006–2007	93.4	88.7	62.6
2009–2010	93.6	88.7	62.8
2012–2013	93.6	91.4	62.8

**Fig. 3:**
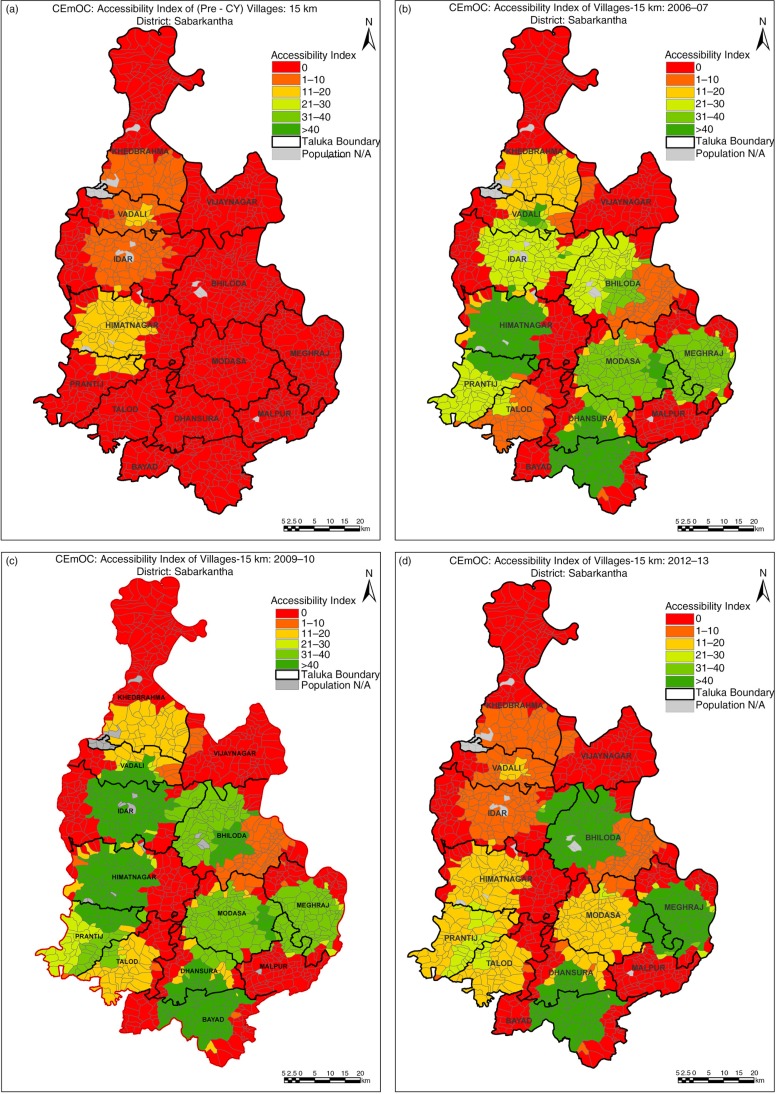
(a–d) Changes in availability in Sabarkantha district.

Sabarkantha showed the maximum increase in average *A*_*i*_ from three beds per 100,000 population in pre-CY period to 23 beds per 100,000 population in 2006. As shown in Fig. 3, it continued to increase to 30 beds per 100,000 population in 2009–2010 to decline by half to 15 beds per 100,000 population in 2012–2013. As in the other districts, availability in previously deprived areas did not change during the study period.

There are several limitations to this study. First, the study includes only three districts of Gujarat, but as the districts are from different regions of Gujarat, the findings are representative. Second, *A*_*i*_ was calculated based on bed capacity of facilities collected in 2012. However, this limitation should not affect the results because the assumption that bed capacity has not changed in the past 6 years is plausible for most facilities. Despite these limitations, the study adds significantly to the existing evaluation of demand-side financing schemes and public–private partnerships, and has implications for similar policies and programs not only in India but also in other developing countries.

## Discussion

This study finding suggests that CY has tremendously increased the geographic availability of free CEmOC services. These findings are consistent with previous literature indicating improved institutional deliveries (41) and increased C-section rates among poor women after implementation of CY (25). Although there was a significant improvement in availability in already served areas, over the period of 6 years from 2006 to 2012, availability did not change significantly for remote areas. Previous literature suggests that there could be clustering of CY providers in towns and absence or limited CY participation by private providers in geographically remote areas (22). Also, the availability did not change over time for remote areas as the public sector facilities from these areas are not providing CEmOC and the private sector participation is negligible. The private providers serving in the geographically remote areas may already have high demand for their services and hence may not have any incentive to participate in government programs such as CY. It is also possible that there were no eligible private practitioners in those areas, and CY was not attractive enough for new providers to set up practice in those challenging areas. The reason for the positive relationship between the number of providers and the average *A*_*i*_ could be the clustering of providers leading to an increased choice in highly served areas.

Poor women from developing countries such as India are more likely to die during pregnancy or related complications as a direct result of lower utilization of maternal healthcare services (42). Since the public healthcare infrastructure in India is weak and there is a dearth of skilled human resources for CEmOC, mothers prefer private sector due to better availability and perceived better quality of care. Public–private partnership for providing CEmOC services is an evidence-based strategy used in many developing countries, including India (43). CY is one of the few demand-side financing schemes involving only private sector providers. Studies examining the change in availability of vital maternal health services such as CEmOC after implementation of such innovative schemes are important to enrich an evidence base for maternal mortality reduction and health systems research.

The study findings also suggest that population-based UN indicators for availability of EmOC are not sensitive enough to capture spatial aspect and hence not sufficient to indicate the ability of the health system to provide optimal EmOC to reduce maternal deaths (15, 16). In Sabarkantha, there were 21 facilities in 2012 providing CEmOC for a population of about 2.4 million (44), which is more than adequate provision of CEmOC as per UN indicators (approximately one CEmOC facility per 100,000 population vs. UN norm of one CEmOC per 400,000 population). Yet, there are areas in this district that have limited access to CEmOC, and women have to travel far to avail lifesaving obstetric services. Literature suggests the need to include physical accessibility as an important determinant to skilled birth attendance, and there is a need to include spatial aspect in determining the availability of CEmOC (45). There is a need for further research and emphasis on adding spatial analysis to get a comprehensive picture of the availability of essential services such as CEmOC.

This paper provides an example of how to use GIS for evaluation of equitable availability and program implementation. Combination of spatial and temporal analyses provides a comprehensive portrayal of current state of the availability of health services including obstetric care.

## Conclusions and program/policy implications

Demand-side financing schemes and public–private partnerships are used globally to improve access to healthcare services, especially maternal healthcare services. Many developing countries have implemented voucher schemes within public and private sectors to increase access to institutional deliveries and EmOC to poor women residing in geographically remote areas. This study shows that the location and number of facilities should be selected based on the location of potential demand, ensuring optimal coverage with the existing resources and avoiding overprovision in already served areas. Needs assessment studies using quantitative methods and GIS techniques such as 2SFCA are recommended to assess demand and supply of available resources along with geographic distribution of healthcare facilities. GIS techniques are an important part of a policy tool kit for effective implementation and monitoring of new programs such as CY. They should be part of impact evaluation methods for providing robust scientific evidence, especially when geographic availability is an important element of the program. This paper adds value not only to health services research but also to the implementation and realistic evaluation research for low-resource settings.
